# Determination of fitness and therapeutic options in older patients with acute myeloid leukemia

**DOI:** 10.1002/ajh.26079

**Published:** 2021-01-12

**Authors:** Jorge E. Cortes, Priyanka Mehta

**Affiliations:** ^1^ Georgia Cancer Center Augusta University Augusta Georgia USA; ^2^ Department of Haematology University Hospitals Bristol, NHS Foundation Trust Bristol UK

## Abstract

Treatment of older patients with AML remains challenging. Although age, performance status, and comorbidities are commonly employed to determine fitness for intensive treatment, several studies have demonstrated improved outcomes with treatment in older and classically unfit patients, highlighting the importance of other disease‐related and patient‐related factors that have prognostic value for treatment outcome in AML. However, consistent and objective assessments for fitness are lacking. Multi‐parameter geriatric assessment tools offer more comprehensive evaluation, but are limited by the required resources and lack of standardization and consensus regarding prognostic value. These assessments are particularly important considering the emerging new AML therapies that represent a spectrum of intensities. Patients should therefore be evaluated holistically for fitness to receive a specific treatment, with the aim of providing individualized care, and such definitions of fitness should also consistently be applied to clinical trials. This review will examine evolving criteria for the determination of fitness among AML patients and discuss treatment options for older and/or unfit patients with AML.

## 1 INTRODUCTION

Acute myeloid leukemia (AML) is the most common adult acute leukemia, accounting for ~80% of cases, with an incidence estimated at 3–5 cases per 100 000 persons in the United States.^1^ AML is primarily a disease of the elderly, with a median age of 68 years at diagnosis.^2^ Historically, the 5‐year overall survival (OS) rate for AML was 29%, but declined to 8% among patients aged ≥65 years.^2^, ^3^

Older age was historically considered a poor prognostic factor and also the main criterion for determining whether an AML patient could receive intensive therapy.^4^ Outcomes among older AML patients treated with conventional induction chemotherapy vary widely, clouding the definition of fitness. The MD Anderson Cancer Center evaluated 446 patients aged ≥70 years who received intensive chemotherapy for AML and found 54% had unfavorable cytogenetics and 31% had a prior malignancy.^5^ Response to intensive chemotherapy included complete remission (CR) in 45%, with an 8‐week mortality of 36%. Median OS was <6 months for all patients and 13.8 months for those achieving CR. The authors concluded that, despite reasonable CR rates, the OS and 8‐week mortality rates did not support intensive chemotherapy for patients aged ≥70 years; however, the study did not include a comparison with non‐intensive regimens.^5^ Similarly, Vey et al reported a CR rate of 43% among AML patients who were aged ≥75 years, with an early mortality rate of nearly 20% and median OS of 9 months.^6^ Results from the Swedish Acute Leukemia Registry update in 2011, which included 998 AML patients aged 70–79 years, indicated lower 8‐week mortality rates with intensive chemotherapy vs palliative treatment, but this population had a lower proportion of patients with high‐risk cytogenetics (proportion of high‐risk cytogenetics in de novo AML: 30%; proportion of high‐risk cytogenetics in secondary AML: 40%).^7^ In a recent, broader analysis from the Swedish AML Registry, which included 6994 AML patients diagnosed between 1997–2016, OS improved significantly over time in those aged 50–75 years, whereas no improvement was seen in younger (<50 years) or older (>75 years) patients.^8^ Overall, 60% of patients received intensive therapy, and patients not receiving intensive therapy had higher early death rates irrespective of age.

As prognostically relevant as age is, there are other factors associated with patient outcomes. Some of these disease‐related and patient‐related characteristics include cytogenetic risk, history of myelodysplastic syndrome (MDS), and comorbidities. Thus, the therapeutic paradigm for older AML patients has been shifting in recent years,^4^, ^9^ with clinicians recognizing the need to assess patients holistically for appropriateness to receive a specific therapy/regimen. However, studies evaluating AML patients vary in design and often have a vague and subjective characterization of fitness for therapy. For example, a recently published study of venetoclax plus a hypomethylating agent (HMA) included patients aged ≥65 years who were ineligible for standard induction chemotherapy, loosely defined as having “various comorbidities, such as age >75 years, cardiac disease or prior anthracycline use, secondary AML, or high probability of treatment‐related mortality.”^10^ These criteria were not defined by objective measures, such as a specific New York Heart Association (NYHA) functional class, ejection fraction, maximum dose of anthracycline, or Chemotherapy Risk Assessment Scale for High‐Age Patients (CRASH) scores. Other studies have been more precise in defining ineligibility to receive intensive therapy, such as the randomized study of glasdegib plus low‐dose cytarabine (LDAC) vs LDAC alone that specified the following criteria: age  ≥75 years, serum creatinine >1.3 mg/dL, severe cardiac disease (left ejection fraction <45%), or Eastern Cooperative Oncology Group (ECOG) performance status (PS) of 2.^11^ The goal of this review is to examine and discuss evolving criteria for the determination of fitness among AML patients and evaluate treatment options for older and/or unfit adults with AML.

## 2 EVOLVING CRITERIA IN THE EVALUATION OF PATIENT FITNESS FOR INTENSIVE THERAPY

### 2.1 Age

Although age should not be a sole determinant of the appropriateness of a patient for intensive therapy, it is appropriate to include age as one of the considerations. Clinical practices related to AML treatment and outcomes were analyzed in AML patients aged ≥66 years in a retrospective cohort study from the Surveillance, Epidemiology, and End Results (SEER) program database and Medicare enrollment and claims files from 2000–2009^12^ Of 8336 eligible patients, 40% received chemotherapy for AML within 3 months of diagnosis. Treatment rates increased from 35% in 2000 to 50% in 2009. Patients receiving treatment had a lower incidence of secondary AML, poor performance indicators (use of oxygen, respiratory supplies, wheelchairs, home health agency services, and skilled nursing facility services), and comorbidities than untreated patients. Treatment reduced the risk for death during the observation period by 33%, with median OS longest among patients treated with intensive therapy (18.9 months) vs HMAs (6.6 months) and no treatment (1.5 months); similar mortality risk reduction was seen in patients aged ≤75 vs >75 years. Factors associated with early death included prior MDS, poor performance indicators, and comorbidities. On the contrary, a large (N = 980), retrospective, single‐center study on AML patients aged ≥70 years diagnosed between 1995–2016 indicated a significant survival benefit with HMAs (median OS = 14.4 months) compared to high‐intensity therapy (10.8 months), low‐intensity therapy (5.9 months), or supportive care (2.1 months).^13^, ^14^ In this study, 37% of patients received high‐intensity therapy, 26% received HMAs, 9% received low‐intensity therapy, and 28% received supportive care; 43% and 57% of patients had de novo AML and secondary AML, respectively. Clinical variables such as secondary AML, poor‐risk cytogenetics, PS, front‐line therapy, age, white blood cell (WBC) count, platelet count, and hemoglobin level at diagnosis were identified as having an impact on OS.^13^, ^14^ These data demonstrate the benefits of AML therapy and illustrate some factors to consider in determining fitness in older patients.

Although there has been less emphasis on age in recent years as a sole determinant of fitness, a retrospective analysis of 968 patients enrolled across five Southwest Oncology Group trials identified frequent correlation between age and other poor‐prognosis factors.^15^ In this early report, published in 2006, older age was associated with a smaller proportion of patients with a PS of 0, relative to younger age. Additionally, the promortion of patients wtih favorable cytogenetics significantly decreased from 17% in younger patients to 4% in patients aged >75 years. There was also a corresponding increase in unfavorable cytogenetics and a higher proportion with multidrug resistance among older patients (57%–62% for ages ≥56 years vs 33% for ages <56 years). Patients with older age and a poor PS had a significantly higher likelihood of 30‐day mortality.^15^

More recently, Lazarevic et al reported clinical and diagnostic features with a focus on patients aged ≥80 years using data from the Swedish AML registry.^16^ Patients aged >85 years had slightly higher WBC counts and blood absolute blast counts, and less elevated lactate dehydrogenase (LDH) levels. Although older patients tended to undergo less morphologic subclassification and genetic evaluation in this study, complex and monosomic karyotypes were more common in this group. Secondary AML was most common in patients aged 70–80 years, but less common in patients aged ≥85 years.^16^ These data suggest modest differences in clinical AML subsets across ages 70–100 years and encourage collection of molecular data in these patients, particularly in the context of emerging therapies, many of which may benefit patients with specific AML subtypes (eg, secondary AML) or molecular features. Studies from the German AML Cooperative Group further underlined the significance of molecular data collection to identify subsets of patients who will most likely benefit from intensive induction therapy. In a study by Metzeler et al in AML patients who received intensive induction therapy, the mutational spectrum in older patients (≥60 years) differed from younger patients (<60 years).^17^ Further, in a study by Prassek et al, among 151 patients aged ≥75 years who received intensive induction therapy, adverse‐risk cytogenetics and isocitrate dehydrogenase 1 (*IDH1*) mutations were identified as negative prognostic factors of OS.^18^

### 2.2 Performance status

Oncology PS measures, such as the ECOG PS or Karnofsky PS (KPS), can aid in identifying higher‐risk AML patients independently of age. Treatment toxicity and 30‐day early mortality are higher in older adults with poor performance scores.^19^ A retrospective analysis assessed outcomes and prognostic factors for 998 patients aged ≥65 years with AML or high‐risk MDS and receiving intensive therapy between 1980–2004.^20^ A multivariate analysis in these patients identified poor ECOG PS (>2) among the prognostic factors associated with CR, 8‐week mortality, and OS.^20^ Importantly, improved supportive care, including the use of prophylactic antibiotics and antifungals in older patients with AML, has helped improve the safety of delivering intensive therapy in older patients.

It is worth noting that PS was developed mostly for evaluating patients with solid tumors. In AML, PS usually refers to function prior to onset of AML‐related symptoms, as factors such as the management of patients prior to the start of treatment (eg, with transfusions, antibiotics, and other supportive care) can make the assessment of PS more challenging and variable. Despite this limitation, ECOG PS and/or KPS have been integrated into most large cooperative group studies evaluating treatment or transplantation of patients with AML.

Several studies have investigated treatment strategies in patients with poor PS. An analysis of 2767 AML patients in the Swedish Acute Leukemia Registry evaluated the effect of the decision to treat on outcomes.^21^ Thus, PS was best in patients aged 40–44 years and declined with increasing age. As PS worsened, the proportion of patients receiving intensive therapy also declined. Thirty‐day mortality rates were dependent on age and PS, but older patients with good PS had low early death rates and patients with poor PS had increased early mortality across all ages. Early death was reported for 36% of patients aged 76–89 years with a PS of 3–4 who were given intensive therapy vs 52% of patients who received palliation only (*P* = .023). While the early mortality rate was higher in patients with impaired PS across age groups, there were some long‐term survivors, suggesting intensive therapy may be of benefit for selected patients.^21^ Among 57 patients with PS of 3–4 treated with intensive cytarabine‐based therapy at MD Anderson Cancer Center, the CR rate was 25% and 8‐week mortality was 77%.^5^ A multivariate analysis found high 8‐week mortality was associated with ECOG PS of 2–4, among other factors.

Together, these studies suggest intensive therapy is superior to low‐intensity therapy, and the latter is superior to supportive care alone in older AML patients, and most patients should be considered for treatment. So, PS is highly linked to age and comorbidities but insufficient alone to accurately assess fitness. Varying degrees of comorbidity, some of which may be optimally managed, in older AML patients highlight the need for better strategies to assess fitness. Thus, more sensitive approaches are needed to better identify candidates for intensive therapy.^4^, ^19^

### 2.3 Comorbidities/medical history

The likelihood of comorbidities increases with age in AML patients and can affect treatment administration and toxicity.^19^, ^22^ Patients with comorbidities are often excluded from clinical studies, limiting data to inform treatment decisions; however, comorbidity indices, such as the Charlson Comorbidity Index (CCI) and the Hematopoietic Cell Transplantation (HCT)–Specific Comorbidity Index (HCT‐CI), have been validated to predict outcomes in AML patients.^23^, ^24^ For example, the HCT‐CI includes objective definitions of comorbidities not only to determine the number of conditions, but also to assess their level of burden.^4^, ^22^ Comorbidities with weighted scores of 3 (highest score) in the HCT‐CI include pulmonary disease (defined by forced expiratory volume in 1 second [FEV_1_] and/or diffusion capacity of carbon monoxide [DL_CO_] ≤65%, dyspnea at rest, or requiring oxygen), hepatic abnormalities (defined by elevations in liver function tests >2.5 × upper limit of normal [ULN] or bilirubin level >1.5 × ULN), heart valve disease (except mitral valve prolapse), and a prior solid tumor.^22^ Among 177 AML patients aged >60 years and treated with induction chemotherapy, those with an HCT‐CI score ≥3 had an early mortality rate of 29% vs 3% and 11% in patients with scores of 0 and 1–2, respectively (*P* <.001).^23^

However, aging and frailty related to aging are not entirely a function of comorbidities. Patients with several well‐managed comorbidities may be reasonably fit and vice versa. Thus, assessment of comorbidities may help better define fitness for intensive therapy, but still does not fully represent the possible outcome and tolerability of treatment for AML patients.^4^, ^19^

### 2.4 Multi‐parameter assessment tools

In response to the somewhat overlapping, yet incomplete, influences of age, PS, and comorbidities to define fitness in AML patients, use of geriatric assessment tools and multi‐parameter assessments has been considered to provide additional prognostic information. Geriatric assessment tools evaluate multiple health domains to more globally assess patient fitness and may assist in refining risk stratification and personalizing therapy for older AML patients^25^; however, there is no consensus, yet on the ideal domains to include and how best to incorporate different factors. Table 1 provides an overview of domains considered in geriatric assessments that have been used in AML patients, and Table 2 summarizes multi‐parameter assessment tools developed from clinical trials.

**TABLE 1:** Geriatric Assessment Tools^66^

Geriatric assessment domain	Tests/tools used
Comorbidity	Charlson Comorbidity Index (CCI) Cumulative Illness Rating Scale–Geriatric (CIRS‐G) Hematopoietic Cell Transplant–specific Comorbidity Index (HCT‐CI) Older Americans Resources Services (OARS) Physical Health Subscale
Cognition	Blessed Orientation‐Memory‐Concentration (BOMC) Mini‐Mental State Examination (MMSE) Modified Mini‐Mental State Examination (3MS)
Depression	Center for Epidemiological Studies–Depression Scale (CES‐D) Geriatric Depression Scale‐15 (GDS‐15) Mental Health Inventory‐17 (MHI‐17)
Distress	Distress Thermometer
Functional status	Activities of daily living (ADL) Eastern Cooperative Oncology Group performance status (ECOG PS) Falls Grip strength Instrumental activities of daily living (IADL) Karnofsky performance status (KPS) Pepper Assessment Tool for Disability (PAT‐D) Short Physical Performance Battery (SPPB) Medical Outcomes Short Form‐36 Health‐related Quality of Life Questionnaire (SF36‐PCS) Timed up and go test Walk speed
Frailty	Fried Frailty Index
Mental health	Medical Outcomes Short Form‐36 Health‐related Quality of Life Questionnaire–Mental Component Score (SF36‐MCS)
Nutrition	Body mass index (BMI) Weight loss
Polypharmacy	Number of medications
Social support	Medical Outcomes Study (MOS) Social Activity Limitations/Social Support Subscales
Quality of life	European Organization for Research and Treatment of Cancer Quality of Life Questionnaire C30 (EORTC QLQ‐C30)

**TABLE 2:** Multiparameter Assessments and Prognostic Models of Fitness for Older Patients With AML

Assessment/reference No. of patients	Factors				Prognostic model
	Age	PS	Comorbidities	Disease characteristics	
Kantarjian 2006^20^ N = 998	≥75 y	ECOG PS >2 (CR, OS), ≥2 (8‐wk mortality)	Creatinine >1.3 mg/dL Antecedent hematologic disorder ≥6 months (CR), ≥12 months (OS)	Unfavorable/complex karyotype WBC ≥25 × 10^9^/L LDH >600 U/L Treatment outside laminar airflow room	Patients with ≥3 factors have CR rates <20%, 8‐wk mortality >50%, and 1‐y survival <10%
Rollig 2010^28^ N = 909	>65 y (3 points)			Cytogenetic risk‐independent risk factor of OS *NPM1* mutation (−2 points) CD34 expression >10% (2 points) WBC >20 × 10^9^/L (2 points)	Four prognostic profiles: Favorable cytogenetics Good intermediate (intermediate cytogenetics and score ≤3) Adverse intermediate (intermediate cytogenetics and score >3) High‐risk cytogenetics
Krug 2010^29^ N = 1406; validation cohort, n = 801	≥60 y			Cytogenetic and molecular risk Body temperature Hb Platelets Fibrinogen LDH De novo vs secondary AML	All patients received IC Calculated scores using disease characteristics, predicted probability of CR and early death
Klepin 2013^26^ N = 74	≥60 y	ECOG PS (≤1 = good; >1 = poor) Physical function: Pepper Assessment Tool for Disability Self‐reported prediagnosis and time of treatment Grip strength SPPB	Cognitive function: 100‐point 3MS exam Depressive symptoms: 20‐item 60‐point CES‐D and HCT‐CI Distress Thermometer: 0–10 point rating	Cytogenetic risk Baseline Hb WBC LDH Prior MDS	OS associated with cytogenetic risk, prior MDS, baseline Hb OS associated with poor cognitive function (3MS <77) and low physical function (SPPB <9) OS was **not** associated with age, ECOG PS, depression, or distress
Deschler 2013^27^ N = 195	≥60 y	KPS (0–100) ADLs (Barthel Index, 0–100, and IADLs, 0–8) Get‐up and Go Test	Charlson Comorbidity Index HCT‐CI Depression: GDS MMSE QOL: EORTC QLQ‐C30	BM blasts % Cytogenetics IPSS in MDS WBC Hb LDH Creatinine Creatinine clearance Albumin	ADL Barthel Index <100; KPS <80, and increased fatigue (≥50 by EORTC QLQ‐C30) were highly predictive of OS regardless of treatment group (BSC, HMA, IC) and similar to disease factors such as poor‐risk cytogenetics and BM blasts ≥20%
Geriatric Assessment in Hematology (GAH) Scale Bonanad 2015,^30^ dela Rubia 2015^31^ N = 349; MDS/AML, n = 116 (33.2%)	≥65 y	Gait speed (<0.8 m/s) ADL (3 items from VES‐13; needs help in ≥1 area) Subjective health status (VES‐13; response of poor or fair)	Number of drugs (≥5) Mood (CES‐D; frequently depressed, 3–7 d/wk) Nutrition: MNA‐SF; ≤8 Mental status: SPMSQ; ≥3 errors Prognostic Index for 4‐Year Mortality in Older Adults, including diabetes, cancer, lung disease, heart failure, BMI, smoking (≥3)		Scale score ranges from 0–8 with 1 point attributed for each dimension based on cutoff point (shown in parentheses) Correlated with ECOG PS and KPS except for the comorbidities domain Increasing GAH score group (≤1, 2–6, >6) was predictive of survival

Abbreviations: 3MS, Modified Mini‐Mental State Examination; ADLs, activities of daily living; AML, acute myeloid leukemia; BM, bone marrow; BMI, body mass index; BSC, best supportive care; CES‐D, Center for Epidemiologic Studies–Depression Scale; CR, complete remission; ECOG, Eastern Cooperative Oncology Group; EORTC QLQ‐C30, European Organization for Research and Treatment of Cancer Quality of Life Questionnaire C30; GDS, Geriatric Depression Scale; Hb, hemoglobin; HCT‐CI, Hematopoietic Cell Transplantation–Specific Comorbidity Index; HMA, hypomethylating agent; IADLs, instrumental activities of daily living; IC, induction chemotherapy; IPSS, International Prognostic Scoring System; KPS, Karnofsky performance status; LDH, lactate dehydrogenase; MDS, myelodysplastic syndrome; MMSE, Mini‐Mental State Examination; MNA‐SF, Mini‐Nutritional Assessment; OS, overall survival; PS, performance status; QOL, quality of life; SPPB, Short Physical Performance Battery; SPMSQ, Short Portable Mental Status Questionnaire; VES‐13, Vulnerable Elders Survey; WBC, white blood cell.

A prospective cohort study evaluated the predictive value of geriatric assessments, including measures of cognitive function, depressive symptoms, distress, physical function, and clinical characteristics, for OS in patients with newly diagnosed AML who were aged ≥60 years and received intensive therapy.^26^ The OS was associated with cytogenetic risk group, prior MDS, and baseline hemoglobin level, but not with age or ECOG PS. Among geriatric assessment measures, poor cognitive function (Modified Mini‐Mental State score <77) and low physical performance (Short Physical Performance Battery score <9) were associated with poor OS and increased the predictive power of the more standard clinical measures by 60%.

Another study examined geriatric and quality‐of‐life assessments in 195 AML and MDS patients aged ≥60 years.^27^ The study measured patient‐related factors, including PS, activities of daily living (ADLs), comorbidities, and disease characteristics (Table 2). Signs of dependence (ADLs <100 and KPS <80) and a fatigue score ≥50 on the European Organization for Research and Treatment of Cancer Quality of Life Questionnaire C30 (QLQ‐C30) provided the strongest prognostic information in the final model beyond the established disease‐related factors of poor‐risk cytogenetics and bone marrow blasts.

In the previously mentioned 2006 study by Kantarjian et al,^20^ a prognostic model was built to predict outcomes in older AML patients using various patient‐related and disease‐related factors to categorize patients into risk groups. Both OS and CR rates were higher in the favorable‐risk and intermediate‐risk groups relative to the unfavorable‐risk group. In a prospective trial of 909 AML patients aged >60 years, prognostic factors that included mutational status were investigated for predictive value on clinical outcomes.^28^ A multivariate analysis determined age, karyotype, *NPM1* mutation status, WBC count, LDH level, and CD34 expression were independent prognostic indicators of OS, and these factors were assigned relative point values (Table 2). Based on the total points and a patient's cytogenetic risk, four prognostic profiles were determined: favorable‐risk cytogenetics, intermediate‐risk cytogenetics with favorable‐risk features (score ≤3), intermediate‐risk cytogenetics with adverse‐risk features (score >3), and high‐risk cytogenetics. The OS for these groups was 40%, 30%, 11%, and 3%, respectively.

Results of cytogenetic analysis to determine risk may not be readily available for AML patients who require immediate treatment. Thus, a web‐based application was used to calculate risk scores from standard clinical and laboratory variables, such as body temperature, age, hematologic measures, LDH level, and AML subtype, with or without knowledge of cytogenetic and molecular risk.^29^ These variables were closely and independently associated with CR and early death, and may assist in making treatment decisions for these patients.

The Geriatric Assessment in Hematology (GAH) scale was designed as a brief evaluation of older patients with hematologic malignancies and consists of eight dimensions of performance, mental status, and health status (Table 2) that contribute to a score of 0–8. It was validated in 349 patients aged ≥65 years with newly diagnosed hematologic malignancies, including AML.^30^ The GAH scale correlated with ECOG PS and KPS, except in the comorbidities domain. Increasing GAH score groups of ≤1, 2–6, and >6 were predictive of survival (*P* <.001).^31^ An abridged geriatric assessment was compared with KPS and the Physical Performance Test in 100 cancer patients aged >70 years, including 14% with hematologic malignancies.^32^ The assessment included some domains consistent with GAH (ADLs, affective status, nutritional status, and polypharmacy), but also considered risk for falls, hearing, vision, urinary incontinence, and pain. Note, OS was associated with the abridged geriatric assessment, but not KPS or the Physical Performance Test score.

## 3 THERAPEUTIC APPROACHES IN OLDER AND/OR UNFIT PATIENT POPULATIONS

Several new therapies have been approved for the treatment of adult AML patients in the past few years, substantially changing the treatment paradigm. Although therapies were traditionally classified as intensive or nonintensive, available therapies now represent a spectrum of intensities. The definition of intensity is also subjective. For example, if the definition is based on myelosuppression, then the depth of myelosuppression (ie, intensity) may be milder and the time to neutrophil and platelet recovery may be faster with the traditional 7 + 3 regimen than with decitabine and venetoclax.^33^, ^34^ Some newer therapies are specifically indicated for use in older and/or unfit patients, but others may also be appropriate for some older patients depending on their overall fitness, thereby expanding treatment options for older patients while avoiding the toxicities posed by conventional chemotherapy.^25^ All patients should thus be assessed for fitness to receive a given therapy or regimen, rather than deemed “fit” or “unfit” overall. Other factors, such as the patient's goals, also need to be included in the treatment decision. The suitability of the treatment administration setting should also be considered; this includes, for example, the ability to administer transfusions for a longer time and to manage septic episodes for therapies with more prolonged myelosuppression. Clinical studies of therapies appropriate for some older AML patients are reviewed below and summarized in Table 3.

**TABLE 3:** Targeted Therapies for AML in Older and/or Unfit Adults: Results of Key Clinical Trials

Study/treatment groups	Patient population	Key efficacy outcomes	Key safety outcomes
**Hypomethylating agents**			
Azacitidine Dombret 2015^37^ Azacitidine, n = 241 vs CCR, n = 247 (BSC, n = 45; LDAC, n = 158; IC, n = 44)	Newly diagnosed de novo or secondary AML >30% blasts Aged ≥65 y (54% were ≥75 y) Cytogenetics: intermediate (65%) or poor risk (35%) ECOG PS ≤2 WBC ≤15 × 10^9^/L Excluded: AML with inv(16)(p13.1q22), t(16;16)(p13.1;q22), t(8;21)(q22;q22), t(9;22)(q34;q11.2)	OS vs CCR: 10.4 vs 6.5 mo (HR = 0.85; *P* = .101) OS vs BSC: 5.8 vs 3.7 mo (HR = 0.60; *P* = .29) CR + CRi vs CCR: 28% vs 25% BSC: 0% LDAC: 26% IC: 48% RBC TI vs CCR: 44% vs 31% Platelet TI vs CCR: 59% vs 43%	Grade 3–4 AEs: febrile neutropenia (28%), neutropenia (26%), thrombocytopenia (24%), pneumonia (19%) 30‐d and 60‐d mortality: 7% and 16% Hospital days for AEs vs CCR: 28.5 vs 38.3 d (*P* <0.001)
Decitabine Kantarjian 2012^38^ Decitabine, n = 242 vs Treatment choice (BSC, n = 28; LDAC, n = 215)	Newly diagnosed de novo or secondary AML ≥20% blasts Aged ≥65 y (71% were ≥70 y) Cytogenetics: intermediate (63%) or poor risk (36%) ECOG PS ≤2 (24% ECOG PS = 2) WBC ≤40 × 10^9^/L Excluded: t(8;21) or inv(16) karyotypes, comorbidities: unstable angina, NYHA class 3/4 CHF	OS vs treatment choice: 7.7 vs 5.0 mo (HR = 0.82; *P* = .037) CR + CRp = 18% vs 8% (OR = 2.5; *P* = .001)	Grade 3–4 AEs vs LDAC: thrombocytopenia (40% vs 35%), anemia (34% vs 27%) 30‐d mortality vs LDAC: 9% vs 8% 60‐d mortality: 19.7% vs 23% for LDAC and 34.5% for BSC
**Venetoclax**			
Venetoclax + LDAC Wei 2019^40^ n = 82	Newly diagnosed de novo or secondary AML (49%) Aged ≥60 y unsuitable for IC due to comorbidity or other factors (not specifically defined) Cytogenetics: intermediate (60%) or poor risk (32%) ECOG PS ≤2 for patients aged ≥75 y and ≤3 for patients aged 60–74 y WBC ≤25 × 10^9^/L Excluded: NYHA class >2 cardiovascular disability	OS: 10.1 mo (95% CI: 5.7–14.2) CR + CRi: 54% (95% CI: 42%–65%)	Grade 3–4 AEs: febrile neutropenia (42%), thrombocytopenia (38%), WBC count decreased (34%) 30‐d mortality: 6%
Venetoclax + LDAC Wei 2020^41^ Venetoclax + LDAC, n = 143 vs LDAC, n = 68	Newly diagnosed de novo or secondary AML (41% vs 34% LDAC) Aged ≥75 y (57% vs 59%) OR Aged 18–74 y and unsuitable for IC due to ≥1 of following criteria: ECOG PS = 2–3, CHF requiring treatment, LVEF ≤50%, chronic stable angina, DL_CO_ ≤65%, FEV_1_ ≤65%, creatinine clearance ≥30 to <45 mL/min, moderate hepatic impairment (bilirubin >1.5 to ≤3.0 × ULN), or other comorbidity precluding IC	OS vs LDAC: 7.2 vs 4.1 mo (HR = 0.75; *P* = .11) CR + CRi vs LDAC: 48% vs 13% (*P* <.001) CR vs LDAC: 27% vs 7% (*P* <.001) TI vs LDAC: 37% vs 16% (*P* <.001)	Grade 3–4 hematologic AEs vs LDAC: thrombocytopenia (45% vs 37%), neutropenia (46% vs 16%), febrile neutropenia (32% vs 29%), anemia (25% vs 22%) Selected serious AEs vs LDAC: febrile neutropenia (16% vs 18%), pneumonia (13% vs 10%), sepsis (6% each), thrombocytopenia (5% vs 3%), anemia (3% vs 0%), and neutropenia (3% vs 0%) 30‐d mortality vs LDAC: 13% vs 16%
Venetoclax + azacitidine or decitabine DiNardo 2019^10^ n = 145	Newly diagnosed AML Aged ≥65 y (36% ≥75 y) Cardiac disease, prior anthracycline, secondary AML (25%), high probability of treatment‐related mortality permitted Cytogenetics: intermediate (51%) or poor risk (49%) ECOG PS ≤2 Excluded: favorable‐risk cytogenetics	OS: 17.5 mo (95% CI: 12.3‐not reached) CR: 37%, CRi: 30% ORR (CR + CRi + PR): 68% Among CRi responders, 34/43 (79%) achieved RBC TI and 40/43 (93%) achieved platelet TI	Grade 3–4 AEs: febrile neutropenia (43%), decreased WBC count (31%), anemia (25%), thrombocytopenia (24%) AEs were generally similar between venetoclax + azacitidine and venetoclax + decitabine No tumor lysis syndrome reported 30‐d and 60‐d mortality: 3%, 8%
**Glasdegib**			
Glasdegib + LDAC Cortes 2019^11^ Glasdegib + LDAC, n = 88 vs LDAC alone, n = 44	Newly diagnosed untreated AML (88%) or high‐risk MDS (12%; blasts: 10%–19%) Aged ≥55 y Cytogenetics: good/intermediate (58%) or poor risk (42%) ≥1 of following criteria: Aged ≥75 y (58%) Serum creatinine >1.3 mg/dL Severe cardiac disease, eg, LVEF <45% ECOG PS = 2 (53%; PS = 0 or 1 eligible if they met ≥1 other inclusion criteria)	OS vs LDAC: 8.8 vs 4.9 mo (HR = 0.51; *P* <.001); there was no difference in OS for 16 patients with MDS (10.9 vs 10.3 mo) CR vs LDAC: 17% vs 2% (*P* <.05) AML patients ORR (CR + CRi + MLFS) vs LDAC: 27% vs 5%	Grade 3–4 AEs vs LDAC: anemia (42% vs 37%), febrile neutropenia (36% vs 24%), thrombocytopenia (31% vs 24%), pneumonia (17% vs 15%) Death due to any AE occurred in 29% vs 42% Abnormal Frederica QTc: 9 patients receiving glasdegib + LDAC and 5 patients receiving LDAC
**IDH1/2 Inhibitors**			
Enasidenib (IDH2 inhibitor) Stein 2017^46^; Pollyea 2017^45^ Relapsed/refractory AML cohort, n = 176; Untreated AML ≥60 y cohort, n = 37	*IDH2*‐mutated AML or MDS with refractory anemia and excess blasts Aged ≥18 y Cytogenetics: intermediate (67%) or poor risk (33%) ECOG PS ≤2	Relapsed/refractory AML patients CR: 19% CRi: 7% ORR (CR + CRi/ CRp + PR + MLFS): 40% OS: 9.3 mo (95% CI: 8.2–10.9) Untreated AML ≥60 y (62% ≥75 y) CR: 19% ORR: 38% OS: 10.4 mo (95% CI: 5.7–15.1)	Among all 239 patients: grade 3/4 AEs: hyperbilirubinemia (12%), IDH‐inhibitor–associated differentiation syndrome (retinoic acid syndrome; 6%), thrombocytopenia (6%), anemia (5%) 30‐d and 60‐d mortality: 5.1% and 13.1%
Ivosidenib (IDH1 inhibitor) DiNardo 2018^48^ Relapsed/refractory cohort, n = 179; Newly diagnosed cohort, n = 28	*IDH1*‐mutated AML Relapsed/refractory cohort: Aged ≥18 y (23% ≥75 y) Cytogenetics: intermediate (59%) or poor risk (28%), missing (13%) ECOG PS ≤2 Secondary AML (33%) Newly diagnosed cohort: Aged ≥75 y (32% <75 y) ECOG PS ≤2 Severe cardiac or pulmonary disease Hepatic impairment (bilirubin >1.5 × ULN) Creatinine clearance <45 mL/min Cytogenetics: intermediate (32%) or poor risk (68%) Therapy‐related AML (11%)	Relapsed/refractory cohort: CR: 25% CRi: 8% CR + CRi: 33% Newly diagnosed cohort: CR: 29% CRi: 14% CR + CRi: 43%	Relapsed/refractory cohort: Grade 3–4 AEs: IDH differentiation syndrome (13%), QT interval prolongation (10%), dyspnea (9%), leukocytosis (8%), tumor lysis syndrome (6%) Newly diagnosed cohort: Grade 3–4 AEs: fatigue (14%), IDH differentiation syndrome (11%), QT interval prolongation (11%), leukocytosis (7%), diarrhea (7%), nausea (7%)
**Gemtuzumab ozogamicin**			
Gemtuzumab ozogamicin Amadori 2016^52^ Gemtuzumab ozogamicin, n = 118 vs BSC, n = 119	Newly diagnosed, untreated de novo or secondary (31%), CD33+ AML Ineligible/unwilling for IC for ≥1 of following criteria: Aged >75 y (64%) Aged 61–75 y with WHO PS score >2 (7%) Cytogenetics: favorable/intermediate (44%) or adverse risk (27%) Serum creatinine and liver function tests ≤1.5 × ULN WBC <30 × 10^9^/L	OS vs BSC: 4.9 vs 3.6 mo (HR = 0.69; *P* = .005) CR + CRi: 27% Clinical benefit rate (CR + CRi + PR + SD lasting >30 d): 57%	Grade 3–4 AEs vs BSC: infection (35% vs 34%), febrile neutropenia (18% vs 24%), bleeding (13% vs 12%), fatigue (12% vs 21%) Death due to any AE: 17% vs 20%
**FLT3 Inhibitors**			
Midostaurin + azacitidine Gallogly 2017^56^ n = 26 (ongoing phase 1/2 study)	Newly diagnosed de novo or secondary (27%) AML *FLT3* mutations not required (no patients had *FLT3*‐ITD mutations) Aged ≥70 y or ineligible for IC ECOG PS ≤2 (PS = 1 in 54%) Adequate hepatic and renal function (≤1.5 × ULN) Complex cytogenetics = 42%	CR: 25% CRi: 6% OS: 262 d (95% CI: 203–472)	Grade 3–4 AEs: infection (35%), febrile neutropenia (15%), hypotension (15%), syncope (15%)
Gilteritinib + azacitidine Esteve 2018^59^ n = 15 (safety cohort to determine dose of gilteritinib to use in combination with azacitidine)	Newly diagnosed FLT3+ AML Ineligible for IC with ≥1 of following criteria: Aged ≥75 y Comorbidities: CHF NYHA class ≤3 or LVEF ≤50% Creatinine >2 mg/dL, dialysis, prior renal transplant Pulmonary disease (decreased DL_co_ and/or oxygen ≤2 L/min) ECOG PS ≥2 Cumulative anthracycline dose >400 mg/m^2^ doxorubicin Hepatic function (bilirubin ≤1.5 × ULN, LFTs ≤2.5 × ULN)	CR: 27% CRi: 40% PR: 13% ORR: 80%	Grade 3–4 AEs: febrile neutropenia (40%), anemia (33%), neutropenia (33%), thrombocytopenia (27%)
Quizartinib Cortes 2019^60^ Quizartinib, n = 245 vs salvage chemotherapy, n = 122	Relapsed/refractory *FLT3*‐ITD AML with (24%) or without HCT Aged ≥18 y (4% ≥75 y) Cytogenetics: favorable (5%), intermediate (74%), or unfavorable risk (10%) ECOG PS ≤2 Adequate hepatic and renal function (bilirubin and creatinine ≤1.5 × ULN, LFTs ≤2.5 × ULN)	OS vs salvage chemotherapy: 6.2 vs 4.7 mo (HR = 0.76; *P* = .02)	Grade 3–4 AEs vs salvage chemotherapy: thrombocytopenia (35% vs 34%), anemia (30% vs 29%), febrile neutropenia (31% vs 21%), neutropenia (32% vs 24%), sepsis/septic shock (19% for both treatment groups), hypokalemia (12% vs 9%)
**CPX‐351**			
CPX‐351 Lancet 2018^33^ CPX‐351, n = 153 vs conventional 7 + 3 IC, n = 156	One of the following AML types: Therapy‐related AML AML with history of myelodysplasia AML with history of CMML De novo AML with karyotypic abnormalities of MDS ECOG PS ≤2 Serum creatinine <2.0 mg/dL Serum total bilirubin <2 mg/dL LFTs <3 × ULN LVEF ≥50%	OS vs 7 + 3: 9.56 vs 5.95 mo (HR = 0.69; *P* = .003) CR + CRi vs 7 + 3: 48% vs 33% (OR = 1.77; *P* = .016) Patients proceeding to HCT vs 7 + 3: 34% vs 25% (*P* = .098)	Grade 3–4 AEs were similar between treatment groups: febrile neutropenia (68%, 71%), pneumonia (20%, 15%), hypoxia (13%, 15%) Median time to neutrophil and platelet recovery in patients who achieved CR + CRi: 35.0 and 36.5 d for CPX‐351 and 29 d for both counts for 7 + 3 30‐d and 60‐d mortality: 6%, 14% for CPX‐351 vs 11% and 21% for 7 + 3

Abbreviations: AEs, adverse events; AML, acute myeloid leukemia; BSC, best supportive care; CCR, conventional care regimen; CHF, congestive heart failure; CI, confidence interval; CMML, chronic myelomonocytic leukemia; CR, complete remission; CRi, complete remission with incomplete neutrophil or platelet recovery; CRp, complete remission with incomplete platelet recovery; DL_co_, diffusion capacity of carbon monoxide; ECOG, Eastern Cooperative Oncology Group; HCT, hematopoietic cell transplantation; HR, hazard ratio; IC, induction chemotherapy; ITD, internal tandem duplication; LDAC, low‐dose Ara‐C (cytarabine); LDH, lactate dehydrogenase; LFT, liver function test; LVEF, left ventricular ejection fraction; MDS, myelodysplastic syndrome; MLFS, morphologic leukemia‐free state; NYHA, New York Heart Association; OR, odds ratio; ORR, overall response rate; OS, overall survival; PR, partial remission; PS, performance status; RBC, red blood cell; SD, stable disease; TI, transfusion independence; ULN, upper limit of normal; WBC, white blood cell; WHO, World Health Organization.

In the clinical management of newly diagnosed AML, immediate treatment start is typically recommended due to poor prognosis. However, more recently, a real‐world analysis from the German Study Alliance Leukemia–Acute Myeloid Leukemia registry arrived at a different conclusion.^35^ In this large analysis of 2263 AML patients who received intensive induction therapy (median age: 59 years; de novo AML: 75%; secondary AML: 15%), time from diagnosis to treatment did not affect the likelihood of response, early death, or long‐term survival. Further, in OS analyses stratified for age ≤60 and >60 years, no significant differences between groups by time from diagnosis to treatment (0–5, 6–10, 11–15, and >15 days) were observed. These findings have direct clinical implications in the context of targeted therapies, suggesting clinically stable patients may benefit from treatment delay to undergo further medical evaluation (ie, genetic and other laboratory test results) before being assigned to the best available treatment option.

### 3.1 Hypomethylating agents

HMAs, including azacitidine (Vidaza; Celgene Corporation, Summit, NJ, USA) and decitabine (Dacogen; Otsuka America Pharmaceutical, Inc., Rockville, MD, USA), achieved superior efficacy compared with best supportive care (BSC) in older adults with newly diagnosed AML who were considered unable to tolerate standard induction chemotherapy in phase 3 clinical trials. Azacitidine and decitabine are approved by the US Food and Drug Administration (FDA) for all MDS subtypes and in combination with venetoclax (see below section) in patients with newly diagnosed AML who are considered unfit for intensive induction therapy. In Europe, azacitidine is approved by the European Medicines Agency (EMA) for AML patients who are not candidates for HCT, and decitabine is approved for patients with newly diagnosed AML who are not candidates for standard induction chemotherapy.^36^

Azacitidine was compared with conventional care regimens, including BSC, LDAC, and intensive chemotherapy, in patients aged ≥65 years with newly diagnosed de novo or secondary AML who had <30% blasts and were not candidates for HCT, with intermediate‐risk or poor‐risk cytogenetics, ECOG PS ≤2, and WBC count ≤15 × 10^9^/L. The patient population encompassed a spectrum of fitness levels; thus, a variety of conventional comparator regimens were available and had to be preselected at the time of randomization.^37^ Azacitidine prolonged OS vs conventional care among patients preselected for BSC (5.8 vs 3.7 months; hazard ratio [HR] = 0.60; *P* = .029), with nonsignificant improvements in patients preselected for LDAC (11.2 vs 6.4 months; HR = 0.90; *P* = .427) or intensive chemotherapy (13.3 vs 12.2 months; HR = 0.85; *P* = .503). Univariate analyses showed favorable trends for azacitidine across all subgroups, including age (<75 vs ≥75 years), ECOG PS of 2, intermediate‐risk and poor‐risk cytogenetics, and prior MDS. Early mortality rates at 30 and 60 days with azacitidine were 7% and 16%, respectively. Patients receiving azacitidine spent fewer days in the hospital for treatment‐emergent adverse events (AEs) vs those receiving conventional regimens.^37^

Decitabine was compared with BSC or LDAC in patients aged ≥65 years with newly diagnosed de novo or secondary AML and characteristics similar to those in the azacitidine trial; although all treatment regimens were lower intensity, the enrollment criteria did not specify patients should have been unsuitable for intensive therapy (Table 3).^38^ Decitabine improved remission rates (CR + CR with incomplete platelet recovery [CRp]: 18% vs 8%; OR = 2.5; *P* = .001) and resulted in a modest but statistically significant improvement in OS (7.7 vs 5.0 months; HR = 0.82; nominal *P* = .037) vs BSC or LDAC. Similarly, OS with decitabine was better in most subgroups, particularly patients aged ≥70 years and those with bone marrow blasts >30%, intermediate‐risk and poor‐risk cytogenetics, and ECOG PS of 2.

### 3.2 Venetoclax

Venetoclax (Venclexta/Venclyxto; AbbVie Inc, North Chicago, IL, USA) is a small‐molecule inhibitor of anti‐apoptotic B‐cell lymphoma 2 (Bcl‐2).^39^ Venetoclax plus LDAC or HMAs was granted accelerated approval by the FDA in 2018 for the treatment of adults with newly diagnosed AML who are aged ≥75 years or have comorbidities precluding the use of intensive chemotherapy, with a requirement for further confirmation of clinical benefit in later‐phase trials.^39^ Venetoclax is not yet approved for AML treatment in Europe,^36^ and there has been limited evaluation of venetoclax in patients who are candidates for intensive chemotherapy.

A phase 1b/2 clinical trial evaluated the safety and preliminary efficacy of venetoclax plus LDAC.^40^ Patients aged ≥60 years with newly diagnosed AML who were deemed ineligible by the investigator for intensive therapy due to comorbidities or other factors were eligible (Table 3); however, comorbidities and other factors precluding intensive therapy were not precisely defined. Patients with NYHA class >2 were excluded, along with those with human immunodeficiency virus infection or with a history of other malignancies. Median OS was 10.1 months and the rate of CR + CR with incomplete recovery of neutrophils or platelets (CRi) was 54% with venetoclax plus LDAC. The 30‐day mortality rate was 6%.^40^ A follow‐up phase 3 trial further evaluated venetoclax plus LDAC in patients aged ≥18 years with newly diagnosed AML who were ineligible for intensive therapy due to age (≥75 years) or ≥1 of the following criteria: ECOG PS of 2–3, history of congestive heart failure requiring treatment, ejection fraction ≤50%, chronic stable angina, DL_CO_ ≤65%, FEV_1_ ≤65%, creatinine clearance ≥30 to <45 mL/min, moderate hepatic impairment with total bilirubin >1.5 to ≤30 × ULN, or any comorbidity thought to preclude the use of intensive therapy.^41^ The median age was 76 years, 32% had poor‐risk cytogenetics, 38% had secondary AML, and 20% had prior HMA exposure. The rate of CR + CRi was 48% with venetoclax plus LDAC vs 13% with LDAC alone, and higher rates of remission were consistently seen with venetoclax plus LDAC across evaluated patient subgroups. However, the trial failed to meet its primary endpoint of improved median OS with venetoclax plus LDAC vs LDAC alone (7.2 vs 4.1 months; HR = 0.75; 2‐sided *P* = 0.11).^41^

A separate phase 1b study evaluated the safety and efficacy of venetoclax plus HMAs (ie, decitabine or azacitidine) in newly diagnosed AML patients aged ≥65 years who were ineligible for standard intensive therapy.^10^ Nearly half of the patients (49%) had poor‐risk cytogenetics. Venetoclax plus an HMA achieved a CR rate of 37% and a CRi rate of 30%, for an overall response rate (ORR = CR + CRi + partial remission) of 68%. The median OS for all patients was 17.5 months. The 30‐day and 60‐day early mortality rates were 3% and 8%, respectively. For secondary AML patients, CR + CRi rate was 67% and median OS was not reached.^10^ A phase 3 trial further evaluated venetoclax plus azacitidine in patients aged ≥18 years with newly diagnosed AML who were ineligible for intensive therapy due to age (≥75 years) or ≥1 of the following criteria: ECOG PS of 2–3, history of congestive heart failure requiring treatment, ejection fraction ≤50%, chronic stable angina, DL_CO_ ≤65%, or FEV_1_ ≤65%.^42^ Among patients who received venetoclax plus azacitidine, the median age was 76 years, 36% had poor‐risk cytogenetics, and 25% had secondary AML. Venetoclax plus azacitidine improved CR + CRi rate (66% vs 28%; *P* <.001) and median OS (14.7 vs 9.6 months; HR for death = 0.66; *P* <.001) vs azacitidine alone. Similarly, OS with venetoclax plus azacitidine was better in most evaluated subgroups, particularly in patients with *IDH1* or isocitrate dehydrogenase 2 (*IDH2*) mutations at baseline.

### 3.3 Glasdegib

Glasdegib (Daurismo; Pfizer Labs, New York, NY, USA) is a small‐molecule inhibitor of the Hedgehog signaling pathway.^43^ Glasdegib plus LDAC was approved by the FDA in 2018 for the treatment of adults with newly diagnosed AML who are aged ≥75 years or have comorbidities precluding the use of intensive chemotherapy^43^; it has not been approved by the EMA.^36^ In the BRIGHT AML phase 2, randomized, open‐label study, glasdegib plus LDAC was compared with LDAC alone.^11^ Eligible patients with newly diagnosed AML or high‐risk MDS were aged ≥55 years and unfit for intensive therapy. The addition of glasdegib to LDAC significantly improved the median OS (8.8 vs 4.9 months with LDAC alone; HR = 0.51; *P* <.001) and the CR rate (17% vs 2%; *P* <.05). Patients with poor cytogenetic risk failed to show a significant difference in OS (4.7 vs 4.9 months, respectively; HR = 0.63; *P* = .064).^11^ A nonrandomized phase 1b arm of the BRIGHT AML study also evaluated glasdegib plus 7 + 3 cytarabine/daunorubicin chemotherapy in 22 adults with newly diagnosed AML or high‐risk MDS; the patient population was a mixture of younger and older adults (median: 59 years [range: 27–70]).^44^ In this population, the CR + CRi rate was 55% (CR rate: 50%) and median OS was 34.7 months.^44^ An ongoing, randomized trial is evaluating whether this combination might be superior to standard chemotherapy alone (ClinicalTrials.gov Identifier NCT03416179).

### 3.4 IDH1/2 inhibitors

Enasidenib (Idhifa; Celgene Corporation, Summit, NJ, USA), a small‐molecule inhibitor of mutated IDH2, was approved by the FDA in 2017 for the treatment of adults with relapsed/refractory *IDH2*‐mutated AML based on results of a phase 1/2 trial of enasidenib monotherapy. Enasidenib is not currently approved for AML treatment in Europe.^36^ A subanalysis of this trial was conducted in 37 older (aged ≥60 years) patients with newly diagnosed AML who were ineligible for intensive therapy and had an ECOG PS ≤2.^45^ The CR + CRi rate was 19%, median OS was 10.4 months, and median event‐free survival was 11.3 months. Among all 239 patients receiving enasidenib, the most common grade 3–4 AEs were hyperbilirubinemia, IDH differentiation syndrome, thrombocytopenia, and anemia.^46^ Tumor lysis syndrome occurred in 8 (3%) patients. Rates of hematologic grade 3–4 AEs were higher among the previously untreated AML patients, with thrombocytopenia in 16% and anemia in 14%.^45^

Ivosidenib (Tibsovo; Agios Pharmaceuticals, Inc, Cambridge, MA, USA), a small‐molecule inhibitor of mutated *IDH1*, was initially approved by the FDA in 2018 for adults with relapsed/refractory *IDH1*‐mutated AML. In 2019, the indication for ivosidenib was expanded to include patients with newly diagnosed *IDH1*‐mutated AML who are aged ≥75 years or ineligible for intensive chemotherapy.^47^ Ivosidenib is not currently approved by the EMA.^36^ Both indications in the United States were based on results of a phase 1, open‐label, single‐arm, multicenter trial of ivosidenib monotherapy. The newly diagnosed AML cohort included patients aged ≥75 years with an *IDH1* mutation who had comorbidities precluding the use of intensive therapy based on ≥1 of the following criteria: baseline ECOG PS ≥2, severe cardiac or pulmonary disease, hepatic impairment with bilirubin level >1.5 × ULN, or creatinine clearance <45 mL/min.^47^ Ivosidenib demonstrated a CR + CRi rate of 43% in patients with newly diagnosed AML; of these patients, 2 (7%) went on to receive HCT.^47^ The most common grade 3–4 AEs in both AML groups were prolonged QT interval on an electrocardiogram and IDH differentiation syndrome. Grade 3–4 fatigue occurred in 14% of patients with newly diagnosed AML, and dyspnea occurred in 9% of patients with relapsed/refractory AML.^48^

Ivosidenib plus azacitidine is also being investigated in a phase 1b/2 open‐label, randomized, multicenter trial in adults with newly diagnosed AML with an *IDH1* mutation who are ineligible for intensive therapy.^49^ Preliminary data included 23 patients, of whom 52% were aged ≥75 years, 26% had secondary AML, and 65% had intermediate‐risk cytogenetics. So, CR with incomplete hematologic recovery (CRh) was defined as CR except for absolute neutrophil count >0.5 × 10^9^/L and platelet count >50 × 10^9^/L. The CR + CRh rate was 65% and ORR (CR + CRi + CRp + morphologic leukemia‐free state + partial remission) was 78%. Kaplan‐Meier–estimated 12‐month OS rate was 82% (95% confidence interval [CI]: 59%–93%).^49^ Ivosidenib plus azacitidine has been granted breakthrough therapy designation by the FDA for adults with newly diagnosed *IDH1*‐mutated AML who are ineligible for intensive therapy.^50^ As with HMAs, venetoclax, and glasdegib, the majority of data reported for enasidenib and ivosidenib in patients with newly diagnosed AML have been in those who are older (eg, ≥75 years) and/or otherwise considered unfit to receive intensive therapy, although criteria vary from study to study.

### 3.5 Gemtuzumab ozogamicin

Gemtuzumab ozogamicin (GO; Mylotarg; Pfizer, Inc., New York, NY, USA) is an antibody‐drug conjugate of an anti‐CD33 antibody with the toxin calicheamicin.^51^ The FDA approved GO in 2017 and the EMA approved it in 2018 as monotherapy or combined with standard cytarabine/daunorubicin chemotherapy for the treatment of adults (ages ≥15 years in Europe) with newly diagnosed, CD33‐positive AML. Also, GO was approved by the FDA as monotherapy for the treatment of patients aged ≥2 years with relapsed/refractory, CD33‐positive AML.^36^, ^51^ A randomized, phase 3 study compared GO monotherapy with BSC in patients aged >60 years with newly diagnosed AML who were deemed ineligible for intensive therapy for ≥1 of the following reasons: aged >75 years, World Health Organization performance score >2 in those aged 61–75 years, or unwillingness to receive standard chemotherapy.^52^ Median OS was 4.9 months with GO vs 3.6 months with BSC (HR = 0.69; *P* = .005), and the CR + CRi rate was 27% among patients receiving GO. The most frequently reported grade 3–4 AEs were infection, febrile neutropenia, bleeding, and fatigue; no GO‐related veno‐occlusive disease was observed. The incidence of AEs leading to death was 17% with GO and 20% with BSC.^52^ However, in a combined analysis of two studies in adults who were deemed ineligible for intensive therapy based on age or fitness, the addition of GO to LDAC improved the CR + CRi rate (30% vs 17% with LDAC alone; OR = 0.48; *P* = .006) but not the 12‐month OS rate (27% vs 25%; HR = 0.99).^53^

### 3.6 FLT3 inhibitors

Midostaurin (Rydapt; Novartis Pharmaceuticals Corporation), a *FLT3* inhibitor with activity against other kinases, was approved by the FDA and EMA in 2017 in combination with cytarabine/daunorubicin chemotherapy for the treatment of adults with newly diagnosed, *FLT3*‐mutated AML (no upper age restrictions).^36^, ^54^ Approval was based on a phase 3 trial in younger adults (aged ≤60 years) with newly diagnosed, *FLT3*‐mutated AML who were fit for intensive therapy. An ongoing phase 1/2 study is evaluating midostaurin plus azacitidine in older adults with newly diagnosed AML who were considered ineligible for intensive therapy.^55^, ^56^ Other eligibility criteria include ECOG PS ≤2 and adequate hepatic and renal function (≤1.5 × ULN). None of the patients had a documented *FLT3* internal tandem duplication (*FLT3*‐ITD) mutation. Among 16 patients evaluable for response, 4 achieved CR and 1 achieved CRi.^56^ Median OS was 262 days (95% CI: 203–472). Grade 3–4 AEs included infection, febrile neutropenia, hypotension, and syncope. Three deaths were reported, all attributed to infection.^56^ A separate phase 1/2 study evaluated midostaurin plus azacitidine in patients with newly diagnosed AML who were not able or refused intensive therapy (24%) and those with relapsed/refractory AML (76%).^57^ Patients were required to have an ECOG PS ≤2 and adequate liver (bilirubin <2 × ULN; alanine aminotransferase ≤2.5 × ULN) and renal function (creatinine <2 × ULN). The CR + CRi rate was 13% and median OS was 22 weeks.^57^

Gilteritinib (Xospata; Astellas Pharma US, Inc., Northbrook, IL, USA), a FLT3 inhibitor, was approved by the FDA in 2018 and the EMA in 2019 for adults with relapsed/refractory *FLT3*‐mutated AML.^36^, ^58^ Approval was based on a phase 3 study of gilteritinib in adults (57% aged <65 years) with relapsed/refractory *FLT3*‐mutated AML. An ongoing, randomized, three‐arm, phase 2/3 study is comparing gilteritinib plus azacitidine vs each agent alone in patients with newly diagnosed, *FLT3*‐mutated AML who were aged ≥75 years or ineligible for intensive therapy.^59^ In addition to age, lack of fitness for chemotherapy is based on the presence of the following comorbidities: congestive heart failure, impaired renal function, ECOG PS ≥2, known pulmonary disease, prior or current malignancy (not requiring concurrent treatment), or prior cumulative doxorubicin exposure >400 mg/m^2^ (or equivalent exposure of another anthracycline). Gilteritinib plus azacitidine achieved a CR + CRi rate of 67%, with 53% having a treatment duration >6 months. The randomized portion of the trial is ongoing.

Quizartinib is a highly potent and selective type II FLT3 inhibitor that moderately inhibits KIT.^60^ The Quantum‐R study was a global phase 3 study comparing quizartinib vs investigator's choice of salvage chemotherapy in patients aged ≥18 years with ECOG PS ≤2 and relapsed/refractory *FLT3*‐ITD primary AML with or without HCT.^60^ The median age was 55 years in the quizartinib group and 57.5 years in the salvage chemotherapy group, with 4% of patients overall ≥75 years of age. Median OS was prolonged with quizartinib vs salvage chemotherapy (6.2 vs 4.7 months; HR = 0.76; *P* = .02); there was no difference in event‐free survival in the intent‐to‐treat population (HR = 0.90; *P* = .11). The most common grade 3–4 AEs were thrombocytopenia, anemia, febrile neutropenia, neutropenia, hypokalemia, and sepsis or septic shock; grade 3 QT prolongation was uncommon with quizartinib.^60^ To date, quizartinib has been approved for salvage therapy of patients with *FLT3*‐mutated AML in Japan, but not in the United States^61^ or Europe. Currently, there are no studies of quizartinib specifically designed for the older, unfit AML population.

### 3.7 CPX‐351

The drug CPX‐351 (Vyxeos; Jazz Pharmaceuticals, Inc., Palo Alto, CA, USA) is a liposomal co‐encapsulation of cytarabine and daunorubicin that delivers a synergistic 5:1 molar drug ratio preferentially to leukemia cells vs normal cells in the bone marrow.^62^ Approval for CPX‐351 was given by the FDA in 2017 and the EMA in 2018 for the treatment of adults with newly diagnosed therapy‐related AML or AML with myelodysplasia‐related changes (AML‐MRC).^36^, ^63^ Approval of CPX‐351 was based on the results of a randomized, phase 3 trial comparing CPX‐351 100 units/m^2^/dose on days 1, 3, and 5 with the conventional 7 + 3 regimen in adults aged 60–75 years with newly diagnosed high‐risk/secondary AML, including those previously treated with HMAs.^33^ Note, CPX‐351 significantly improved the median OS (9.56 vs 5.95 months; HR = 0.69; 1‐sided *P* = .003) and remission rate (CR + CRi; 48% vs 33%; OR = 1.77; two‐sided *P* = .016) vs 7 + 3. Further, improved outcomes were seen with CPX‐351 vs 7 + 3 among both patients aged 60–69 years (median OS: 9.63 vs 6.87 months; CR + CRi: 50% vs 36%) and those aged 70–75 years (median OS: 8.87 vs 5.62 months; CR + CRi: 44% vs 28%). More patients in the CPX‐351 vs the 7 + 3 arm proceeded to HCT (34% vs 25%), and median OS landmarked from the date of HCT was longer with CPX‐351 (not reached vs 10.25 months; HR = 0.46; one‐sided *P* = .009). The safety profile of CPX‐351 was generally consistent with that of 7 + 3, including the types, frequencies, and severities of AEs. Among patients who achieved CR + CRi, median time to recovery of neutrophil and platelet counts was longer with CPX‐351 vs 7 + 3. Early mortality rates at 30 and 60 days were lower with CPX‐351 vs 7 + 3, although the difference did not reach statistical significance.^33^

Although CPX‐351 has primarily been studied as intensive chemotherapy, a phase 2 study evaluated lower‐intensity doses of CPX‐351 in adults with newly diagnosed AML who were considered less fit and had a composite treatment‐related mortality score of >13.1 (corresponding to a >13.1% probability of death within 28 days of receiving intensive chemotherapy).^64^ Among patients who received CPX‐351 32 units/m^2^/dose (n = 38) and 64 units/m^2^/dose (n = 10), respectively, the CR + CRi rates were 29% and 20%, 12‐month OS rates were 17% and 20%, and early mortality rates within 28 days were 29% and 40%.^64^ An ongoing clinical trial is also evaluating lower‐intensity CPX‐351 plus venetoclax in adults considered unfit for intensive therapy (ClinicalTrials.gov Identifier NCT04038437).

## 4 PRACTICAL GUIDANCE AND ADDITIONAL CONSIDERATIONS FOR IDENTIFYING APPROPRIATE THERAPIES FOR OLDER PATIENTS

A significant proportion of older AML patients are not offered chemotherapy because of the perceived lack of efficacy and toxicity of intensive chemotherapy. As a result, prognosis in this patient population remains poor.^12^, ^25^, ^65^ While the need for a more holistic approach to determining specific therapies and regimens is recognized, proper objective fitness assessments have traditionally been lacking.

Results of multi‐parameter assessments and prognostic models have led to several important findings: (a) disease‐related factors of unfavorable cytogenetics and multidrug resistance increase with age and are consistently associated with poorer outcomes; (b) prior MDS, percentage of blasts, WBC count, LDH level, and cytogenetic and molecular aberrations may play a role in defining prognosis; (c) increasing age, poor PS, and comorbidities correlate with poorer AML outcomes, but are insufficient to fully assess patient fitness on their own; (d) patient‐related factors, including physical status/frailty, cognitive status, psychologic status, nutritional status, functionality (instrumental ADL), and ability to perform ADLs, may predict OS. These assessments, however, can be time consuming, and additional evidence of their predictive ability for individual therapies/regimens are needed, specifically in older AML patients.

Each patient should be assessed for fitness to receive particular therapies/regimens and other considerations that might inform treatment decisions (eg, molecular targets, patient's treatment goals, logistics), with the aim of providing individualized care. Newer treatments with different safety profiles are generally better tolerated than conventional chemotherapy and may be preferable options for older and unfit patients. In many instances, these drugs have been specifically investigated and/or approved for segments of this patient population.

## 5 CONCLUSIONS

In addition to age, PS, cytogenetic risk category, and AML subtype (de novo, secondary) play a role in defining prognosis in older AML patients. There are several approaches for determining fitness in older AML patients; however, more consistent and objective criteria are needed for classifying patient fitness. The assessment and definition of comorbidities are variable across trials, but cardiac disease and renal impairment as measured by elevated serum creatinine are consistently used to define unfit populations. Several multi‐parameter/geriatric assessment tools are in development to provide a more complete and objective assessment of patient fitness; however, there is no consensus on the most important parameters to include, and interventional clinical trials are not yet using geriatric assessments or assessing individual cognitive, psychologic, or physical status to determine eligibility.

Newer therapies offer varying degrees of treatment intensity and may be appropriate for different subsets of older patients, depending on their overall health, treatment goals, and other considerations. Studies of new therapies have demonstrated improved outcomes in older and classically unfit patients, re‐emphasizing the importance of re‐evaluating the definition of fitness and individualizing treatment strategies.

## CONFLICT OF INTEREST

Jorge E. Cortes has competing financial interests that include consulting fees from Agios, Astellas Pharma, Daiichi Sankyo, Jazz Pharmaceuticals, Novartis, and Pfizer; and institutional research funding from Arog, Astellas Pharma, Daiichi Sankyo, Jazz Pharmaceuticals, Novartis, and Pfizer. Priyanka Mehta has received consulting fees from Daiichi Sankyo, Jazz Pharmaceuticals, Novartis, and Pfizer.

## Data Availability

All relevant data are provided within the manuscript.
